# Conservation and divergence of metabolic phenotypes between patient tumours and matched xenografts

**DOI:** 10.1038/s42255-025-01338-2

**Published:** 2025-07-29

**Authors:** Aparna D. Rao, Ling Cai, Marelize Snyman, Rachel E. Walsdorf, Xiangyi Li, Sophia N. Wix, Gabrielle Gard, Ariel B. Brown, Juliana Kim, Joao Santos Patricio, Sarah Muh, Misty Martin Sandoval, Lauren G. Zacharias, Kristen A. Heimdal, Wen Gu, Jade Homsi, Brittny Tillman, Rohit Sharma, Travis W. Vandergriff, Ashley Solmonson, Brandon Faubert, Thomas P. Mathews, Sean J. Morrison, Ralph J. DeBerardinis, Jennifer G. Gill

**Affiliations:** 1 Children’s Medical Center Research Institute, University of Texas Southwestern Medical Center, Dallas, TX USA; 2 Peter MacCallum Cancer Centre, Melbourne, Victoria Australia; 3 Sir Peter MacCallum Department of Oncology, The University of Melbourne, Melbourne, Victoria Australia; 4 Quantitative Biomedical Research Center, Peter O’Donnell School of Public Health, University of Texas Southwestern Medical Center, Dallas, TX USA; 5 Department of Dermatology, University of Texas Southwestern Medical Center, Dallas, TX USA; 6 Department of Medical Oncology, University of Texas Southwestern Medical Center, Dallas, TX USA; 7 Department of Otolaryngology, University of Texas Southwestern Medical Center, Dallas, TX USA; 8 Department of Surgical Oncology, University of Texas Southwestern Medical Center, Dallas, TX USA; 9 Green Center for Reproductive Biology Sciences, University of Texas Southwestern Medical Center, Dallas, TX USA; 10 Section of Hematology and Oncology, Department of Medicine, University of Chicago, Chicago, IL USA; 11 Howard Hughes Medical Institute, Chevy Chase, MD USA; 12 Department of Pediatrics, University of Texas Southwestern Medical Center, Dallas, TX USA; 13 Eugene McDermott Center for Human Growth and Development, University of Texas Southwestern Medical Center, Dallas, TX USA

**Keywords:** Cancer metabolism, Cancer models, Melanoma, Metabolism

## Abstract

Patient-derived xenografts (PDXs) are frequently used as preclinical models, but their recapitulation of tumour metabolism in patients has not been closely examined. We developed a parallel workflow to analyse [U-^13^C]glucose tracing and metabolomics data from patient melanomas and matched PDXs. Melanomas from patients have substantial TCA cycle labelling, similar to levels in human brain tumours. Although levels of TCA cycle labelling in PDXs were similar to those in the original patient tumours, PDXs had higher labelling in glycolytic metabolites. Through metabolomics, we observed consistent alterations of 100 metabolites among PDXs and patient tumours that reflected species-specific differences in diet, host physiology and microbiota. Despite these differences, most of nearly 200 PDXs retained a ‘metabolic fingerprint’ largely durable over six passages and often traceable back to the patient tumour of origin. This study identifies both high- and low-fidelity metabolites in the PDX model system, providing a resource for cancer metabolism researchers.

## Abstract (web summary)

Rao and Cai et al. perform a detailed metabolic comparison between primary tumours from patients and their matching xenografts, which identify conserved as well as divergent metabolic patterns.

## Main

In the past decade, the PDX model system has emerged as an important tool for studying cancer biology^1^. PDXs have been lauded for their recapitulation of human cancer genetics^2^, heterogeneity^3^, therapy response^4^ and metastatic capacity^5^. In recent years, the PDX model system has also been used to dissect the biology of tumour metabolism in vivo in many cancer types^6^, including melanoma^7–9^, and obtain preclinical data for drugs targeting putative metabolic vulnerabilities. However, the fidelity in translating tumour metabolism across host species remains an important open question^10^, particularly because PDXs are grown in murine hosts, which differ in their immune status, colonizing microbiota, species-specific metabolism and diet. To date, limited studies have explored how well PDXs reproduce the original patient’s tumour metabolism^11,12^. In this study, we developed a parallel workflow to directly compare the metabolism of tumours from patients with melanoma and their matched PDXs using metabolomics and in vivo [U-^13^C]glucose isotope tracing. Although we identify a subset of metabolites and pathways altered during xenotransplantation, most PDX tumours maintain a unique and durable ‘metabolic fingerprint’ that traces back to the original patient and distinguishes them from other PDXs. Here, we provide a characterization of pathways and metabolites, ranked by their fidelity and durability after xenotransplantation, providing a resource for cancer-metabolism researchers.

## Results

**Fig. 1:**
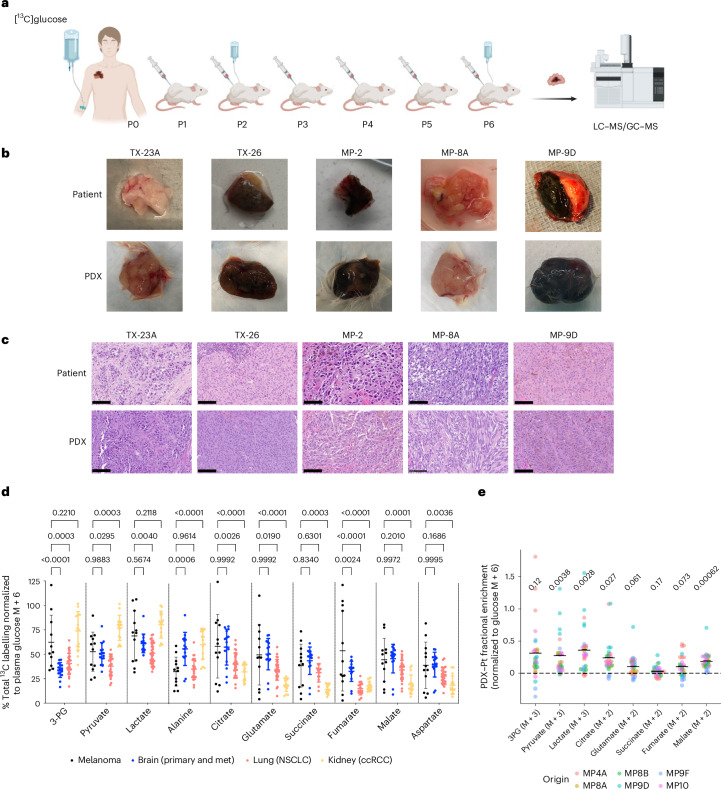
In vivo labelling of human melanoma and matched PDXs infused with [U-^13^C]glucose. **a**, Schematic of a workflow for parallel metabolic analysis of melanomas in patients (P0) and PDXs carried through multiple passages (P1–P6) in mice. LC–MS/GC–MS, liquid chromatography–mass spectrometry and gas chromatography–mass spectrometry. Created using BioRender. **b**,**c**, Representative photographs (**b**) and histology images (**c**) of tumour samples from patients and matched early passage PDXs. Scale bar = 100 µm. **d**, Total labelling (1 – (M+0)) of indicated metabolites as analysed by mass spectrometry and normalized to [^13^C]glucose enrichment (M+6) in patient plasma. Melanoma tumours (*n* = 12, from 6 patients) from this study were compared with published data from brain^16^, lung^14^ and kidney cancer^16^. Data are mean ± s.d. Statistical analysis was done using two-way analysis of variance (ANOVA) with Tukey’s post hoc test; *P* values are shown. NSCLC, non-small-cell lung cancer; ccRCC, clear cell renal cell carcinoma; met, metastasis. **e**, Labelling of indicated metabolites normalized to [^13^C]glucose tumour enrichment, displayed as the absolute difference between the patient (Pt) and P2 or P3 PDXs (*n* = 27). Each data point represents an individual PDX tumour; midlines mark the mean. *P* values are shown above each plot for reference, indicating variations in metabolite labelling due to the host type in the two-way ANOVA. Source data

To evaluate the metabolic phenotypes of tumours from patients with melanoma and their matched PDXs, we developed a protocol to obtain fresh melanoma samples during standard-of-care surgical resection. A portion of melanoma tissue was snap-frozen for metabolomics analysis, and an adjacent portion was digested and injected subcutaneously into immunocompromised NSG mice for PDX formation. A subset of patients received intraoperative infusion of glucose uniformly labelled with ^13^C (U-^13^C) prior to resection to enable further metabolic analysis through isotope tracing. Our isotope infusion approach is similar to published protocols used to assess fuel utilization in human brain^13^, lung^14,15^, kidney^16^ and paediatric^17^ tumours. To generate PDXs, we used a well-established method that recapitulates important aspects of melanoma biology, including metastasis^5,7,18,19^. In addition to comparing metabolic features of patient samples and matched PDXs, we also serially passaged the PDXs (up to six generations) because most pre-clinical studies use PDXs already subjected to several passages in mice (Fig. 1a).

Data and PDXs were derived from 33 melanoma samples from 24 patients (Supplementary Table 1). Most patients had not received any prior treatment and had stage III or IV melanoma, and 16 of the tumours had a *BRAF*^V600E^ genotype. Most samples were collected from regional lymph-node metastases, although primary melanomas and distant metastases were also obtained. From the 33 samples, we generated 23 PDXs, yielding an engraftment rate of 70% (Supplementary Table 1). Engraftment from lymph-node samples (56%) was lower than that from primary (90%) and distant metastases (80%), and graft-versus host disease (GVHD) was observed in 39% of the lymph-node subgroup. The PDXs frequently retained gross macroscopic and histologic features observed in the original patient samples (Fig. 1b,c).

**Extended Data Fig. 1:**
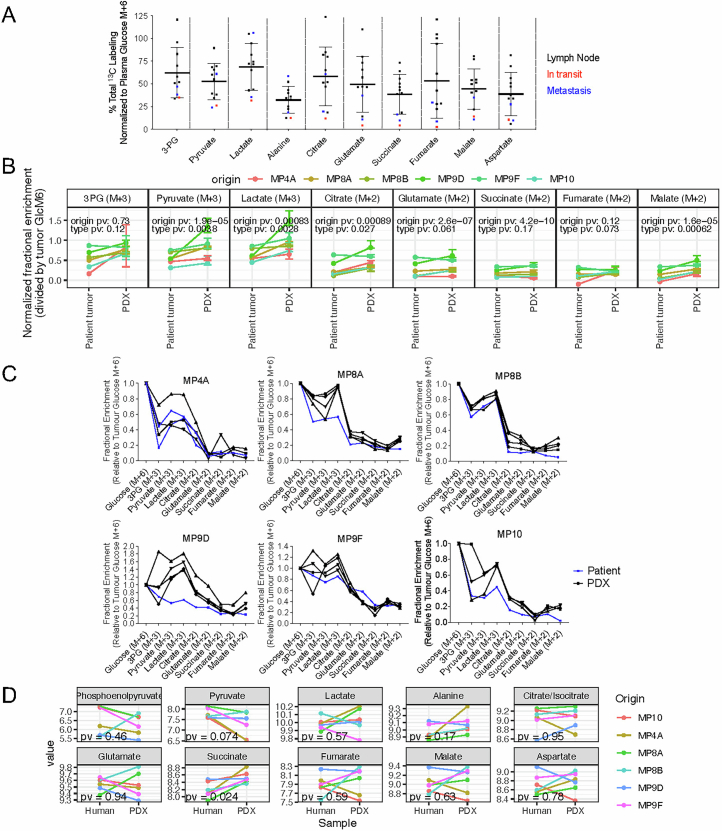
^13^C enrichment in early passage PDX relative to patient tumors. (**a**) Total labeling [1-(M + 0)] of indicated metabolites in melanoma patient tumors normalized to ^13^C-glucose enrichment (M + 6) in plasma (See Fig. 1d, n = 12, mean ± SD). Note that the samples are colored by the site of tissue procurement, with no obvious difference by site. (**b**) Each plot shows the ^13^C enrichment of selected labelled markers normalized to tumor Glucose M + 6, with results for patient tumors (n = 6) and their corresponding PDX samples (n = 27) plotted for comparison, error bars represent upper and lower quartiles. Lines connect samples of the same patient origin, from patient tumor to median of PDX, and each origin type is denoted by a different color. Statistical significance between origins and tumor types (P-values) are shown above each plot for reference, indicating variations in metabolite labeling due to the origin of the PDX lines and the host type in the two-way analysis of variance (ANOVA). (**c**) Fractional enrichment of indicated metabolites normalized to tumor Glucose M + 6. Note that PDX samples (black, n = 3-4) exhibit higher glycolytic intermediate labeling than the parental patient tumors (blue). (**d**) Comparison of relative metabolite abundance between tumors and matched PDXs (6 pairs). P-values from two-sided paired t-tests are provided in each plot.

Because melanoma metabolism has not been characterized in patients through in vivo isotope tracing, we first compared how melanomas and other human tumours utilize glucose. Six patients were infused with [U-^13^C]glucose, and ^13^C enrichment of metabolites extracted from 12 melanoma samples was analysed. First, in metabolites of interest, we assessed total ^13^C enrichment (that is, 1 – (M+0)) normalized to enrichment in plasma glucose. This allowed us to compare glucose metabolism between melanomas and other human tumours analysed in a similar manner^16^. Relevant labelling data from tumours and plasma are provided in Supplementary Tables 2 and 3. The initial analysis revealed a high degree of labelling in glycolytic and tricarboxylic acid (TCA) cycle intermediates in the melanoma samples. Metabolites related to the TCA cycle (citrate, glutamate, succinate, fumarate, malate and aspartate) were labelled as extensively in melanomas as in any other tumour type (Fig. 1d). Although only a small number of patients with melanoma received the infusion, the melanoma samples displayed the broadest labelling range of all cancers studied so far, possibly reflecting a particularly high level of metabolic heterogeneity in this cancer type. This does not seem to stem from differences in tumour site, because most samples came from lymph nodes, which spanned the full range of ^13^C enrichment levels (Extended Data Fig. 1a).

**Extended Data Fig. 2:**
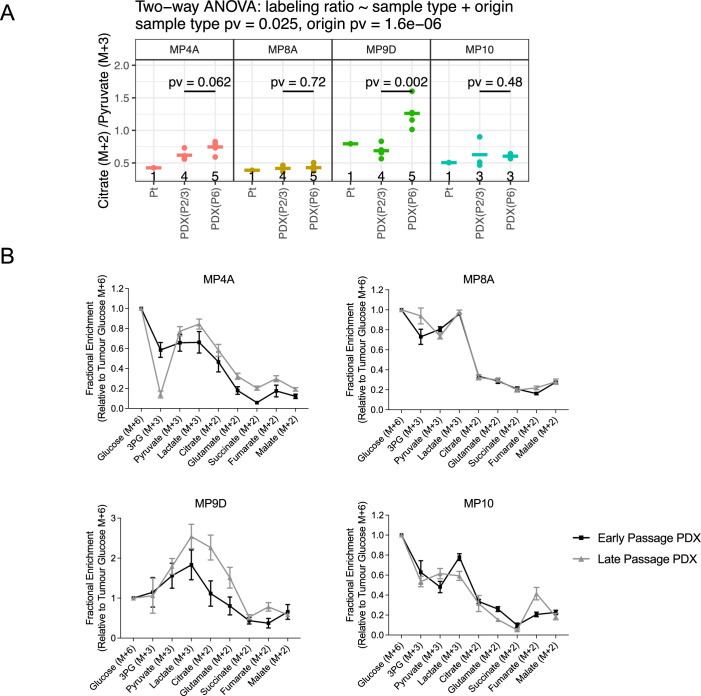
^13^C enrichment in early and late passage PDX relative to patient tumors. (**a**) Four matched sets of citrate m + 2/pyruvate m + 3 ratios are shown for tumors from: patient, early passage PDX, and late passage PDX. PDX data are mean ± SEM (with each data point representing a mean of 3 values from an individual PDX tumor), n = 3-5, with individual n detailed on the x-axis. P-values were calculated using two-way ANOVA, modeling the labeling ratio as a function of sample type and origin. (**b**) Each plot shows the ^13^C enrichment of selected metabolites normalized to tumor Glucose M + 6, with results for early passage (P2 or P3, shown in black) and late passage (P6, shown in grey) PDXs. PDX data are mean ± SEM, with n as follows: MP4A (early passage n = 4, late passage n = 5); MP8A (early passage n = 4, late passage n = 4); MP9D (early passage n = 4, late passage n = 5); MP10 (early passage n = 5, late passage n = 3).

To compare ^13^C labelling features between patients and PDX models, we infused PDX-bearing mice (six PDX lines generated from four patients) with [U-^13^C]glucose. We calculated the difference in normalized fractional enrichment of the major labelled forms of glycolytic and TCA cycle intermediates between the PDX and patient samples. Notably, this analysis included both early-passage (P1 or P2) and late-passage (P6) PDXs. We observed an excess of labelling in glycolytic intermediates, pyruvate and lactate in nearly every PDX sample (Fig. 1e and Extended Data Fig. 1b,c). These discrepancies could not be attributed to differences in pool sizes, because the abundances of these metabolites were similar in patient and PDX tumours (Extended Data Fig. 1d). In contrast to metabolites linked to glycolysis, the fractional enrichment of those related to the TCA cycle was very similar in patient and PDX samples (Fig. 1e and Extended Data Fig. 1b,c). The citrate M+2/pyruvate M+3 ratio is used as a surrogate of the contribution of pyruvate dehydrogenase (PDH) to TCA cycle labelling and has been associated with metastasis in human kidney^20^. This ratio was conserved between patients and PDXs (Extended Data Fig. 2a) aside from one late-passage PDX cohort (MP9D p6), which exhibited wide variation in citrate labelling (Extended Data Fig. 2b).

**Fig. 2:**
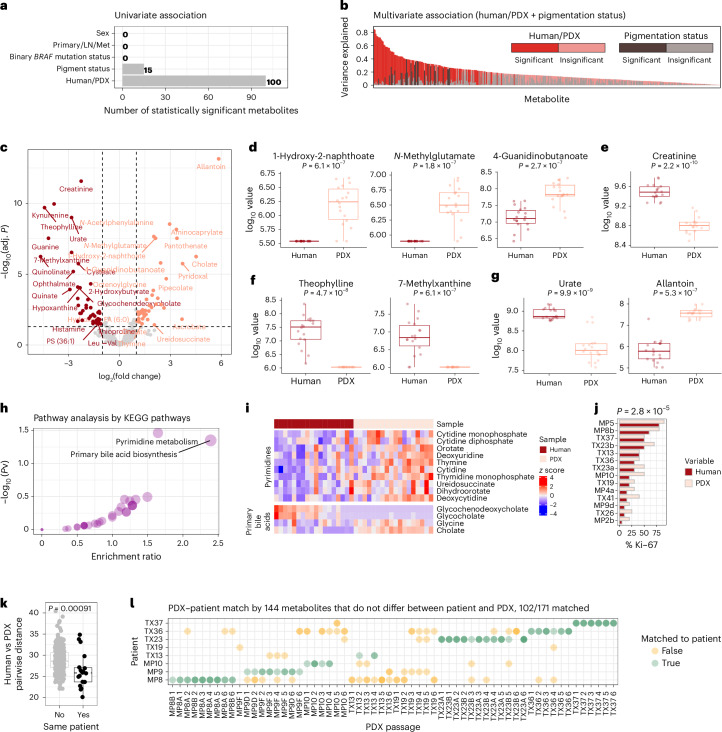
Patient tumour xenotransplantation is associated with characteristic metabolic alterations. **a**, Univariate association between metabolite levels and tumour type or clinical features. Primary/LN/met, primary, lymph node or met; human/PDX, human or PDX. **b**, Partition of variance explained in a multivariate model predicting metabolite levels from human/PDX sample type and pigmentation status. **c**, Volcano plot of metabolites exhibiting different levels in patient and paired P1 PDXs. **d**–**g**, Selected metabolites with prominent differences between patient and PDX, including microbiota-derived metabolites (**d**), dietary metabolites (**f**) and physiology-specific metabolites (**e**,**g**). **h**, Pathway analysis of altered metabolites. **i**, Heatmap of altered metabolites in enriched pathways. **j**, Percentage KI-67 staining compared between patient tumours and PDXs of the same origin. The indicated *P* value was obtained through a two-sided paired *t*-test. **k**, Pairwise Euclidean distance between human tumours and PDXs from the same or different patients, calculated on the basis of all metabolites. **l**, Matching PDX to patient by minimal pairwise Euclidean distance from 144 species-agnostic metabolites. For **d**–**g** and **k**, central lines indicate the median value, and boxplots represent the interquartile range (IQR) with whiskers extending to the smallest and largest values within 1.5 times the IQR. Mann–Whitney tests were performed to calculate *P* values, as described in Methods. *n* = 36 (18 pairs of matched patient tumour and PDX) for **a**–**i**; *n* = 28 (14 pairs) for **j**; n = 199 (11 patient tumours and 188 PDXs for **k** and **l**, see also Extended Data Figure 3c for details).

To test whether tumour metabolomic profiles are maintained after PDX generation, we analysed metabolomic profiles in 18 melanoma patient (P0) samples, alongside matched early passage (P1) PDX samples. An initial univariate analysis was performed to identify the clinico-pathologic factors with the greatest impact on metabolic variance across all samples. Host species (that is, human versus mouse) was the most prominent factor, with 100 differentially expressed metabolites (Fig. 2a and Supplementary Table 4). Tumour pigmentation status revealed that levels of 15 metabolites were altered, whereas other features, such as *BRAF* mutation status, initial site of metastasis and patient sex, did not contribute to metabolic variance in these models (Fig. 2a and Supplementary Table 5). In a multivariate model, we identified some metabolites that were influenced by both host and pigmentation status, reflecting contributions from multiple factors (Fig. 2b).

Because host species played a significant role in metabolic variation, we next sought to identify metabolites enriched in either PDXs or patients (Fig. 2c). Host-specific metabolites fell into several categories, including microbial, dietary and species-specific pathway metabolites. Mouse-specific microbiome-derived metabolites, including 1-hydroxy-2-napthoate, *N*-methylglutamate and 4-guanidinobutanoate, were found to be enriched in PDX tumours, highlighting the influence of host microbiota (Fig. 2d). We also identified metabolites related to the differences in endogenous metabolism and diet between mice and humans. Creatinine levels were higher in human tumours, reflecting the established difference in circulating levels between the two species^21,22^ (Fig. 2e). Dietary influences included theophylline and 7-methylxanthine, two caffeine derivatives that were among the most differentially abundant metabolites in humans (Fig. 2f). We also observed a higher abundance of urate and a lower abundance of allantoin in patient tumours, reflecting the presence of urate oxidase in mice but not in humans^23^ (Fig. 2g).

We performed a broader pathway analysis to identify altered pathways in PDXs (Fig. 2h) and found that levels of components of pyrimidine metabolism were higher in PDXs than in patient tumours (Fig. 2i). Interestingly, a similar phenomenon, noted 40 years ago in enzymatic assays comparing human colon carcinomas and xenografts, was attributed to the faster growth of xenografts^24^.

**Extended Data Fig. 3:**
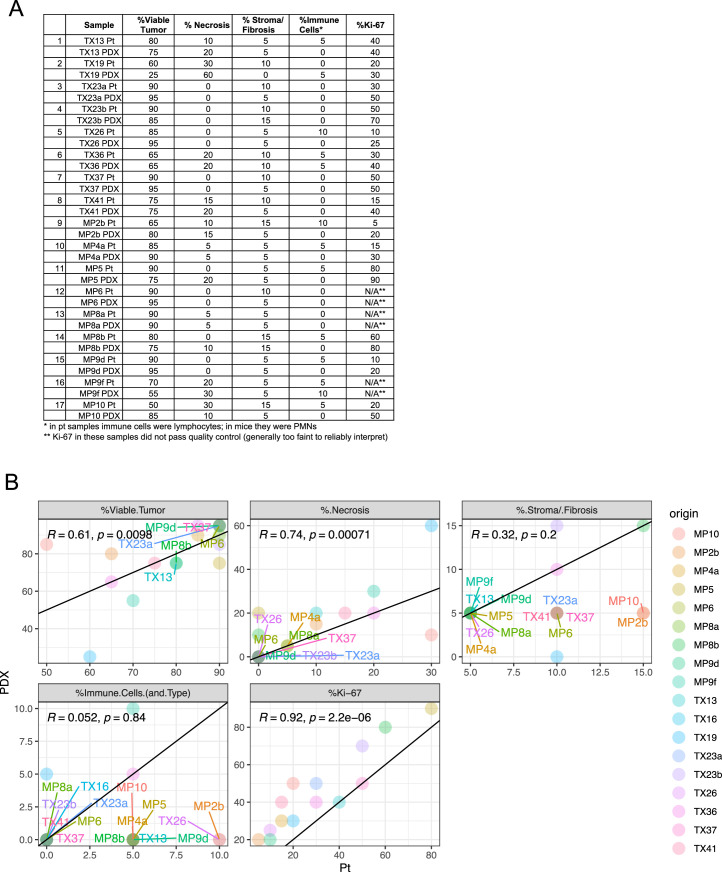
Tumor and PDX pathology analysis. (**a**) Quantification of 17 pairs of matched patient/PDX tumors for markers of histology, immune composition, and proliferation. (**b**) Visualization of comparison of patient tumor pathology features (x-axis) and PDX pathology features (y-axis) by scatterplot with Pearson correlation statistics (N = 18 pairs).

To gain further insight, a dermatopathologist assessed histologic features of 17 of our matched patient and PDX tumours for microenvironmental composition and Ki-67 proliferation index. Overall, tumours were histologically similar between patient and PDX tumours, with a few key differences noted (Extended Data Fig. 3a,b). Matched tumours had strong correlations in the percentage of viable tumour (*r* = 0.61) and necrosis (*r* = 0.74). Generally around 5% of the tumour bed consisted of stromal cells and/or fibrosis. This percentage was similar in about half of the pairs and differed modestly in the others (*r* = 0.32). The percentage of the tumour bed composed of immune cells was very different between patient and PDX samples (*r* = 0.052), as expected, because the PDXs are grown in immunodeficient (NSG) mice that lack lymphocytes. Patient tumours generally comprised ≤10% immune cells, with lymphocytes always being the predominant cell type. PDX tumours rarely had inflammatory infiltrates; when they did, the infiltrate consisted of polymorphonuclear leucocytes (PMNs), such as neutrophils. Despite these histological discrepancies, the low overall contribution of stromal and immune cells means that they are unlikely to be predominant drivers of differences in metabolite levels between patient tumours and PDXs.

PDXs nearly always had higher Ki67 proliferation indices (Fig. 2j), with a median relative proliferation index of 150% (range, 100–400%) compared with the matched patient tumour. Notably, despite these differences, the relative proliferation rates across patients were highly conserved (*r* = 0.92, *P* = 2.2 × 10^−6^) (Extended Data Fig. 3b) indicating the most proliferative tumours in patients were the most proliferative in NSG mice. The increased proliferation observed in PDXs might result from decreased host immunity, a selective advantage of proliferative clones during engraftment or both. From all the histologic data examined, this increased proliferation appears to be the most likely driver of the alterations in pyrimidine synthesis following engraftment.

In addition to pyrimidine synthesis, primary bile acid pathways were also altered in PDXs (Fig. 2h,i). In mice, the enzyme responsible for amino acid conjugation of bile acids is specific to taurine, leading to predominantly taurine-conjugated bile acids. By contrast, around 75% of bile acids in humans are glycine conjugated (that is glycocholate and glycochenodeoxycholate)^25^, and circulating levels of these intermediates are higher in humans than in mice^26^.

Despite species-specific differences, we wanted to know whether PDX tumours retained metabolic features unique to the human tumour from which they were derived. Gauging similarity by Euclidean distance of the *z*-transformed metabolite levels, we observed that pairwise comparisons of human and PDX samples from the same patient were more similar than were samples from different patients (Fig. 2k). This indicates that there is a substantial degree of metabolic durability after xenotransplantation. To test whether this fidelity was maintained after serial passaging, we performed liquid chromatography and mass spectrometry (LC–MS) metabolomics on 171 melanoma samples, including 11 tumours collected from 8 patients, passaged for 6 generations. We then asked whether metabolomic profiles could be used to predict which patient tumour gave rise to each PDX sample. Using 144 metabolites that do not differ between PDXs and patient tumours (*P* > 0.1), most PDX samples (102/171) could be traced back to their patient tumour of origin (Fig. 2l). Several PDXs (MP8A, MP8B, TX23A, TX23B, TX36 and TX37) nearly always matched the original patient tumour; one PDX (TX19) virtually never matched; and the rest (MP9D, MP9F, MP10, and TX13) matched often but displayed infidelity in some samples. Thus, 10 of 11 PDX tumours frequently traced back to the patient tumour.

**Extended Data Fig. 4:**
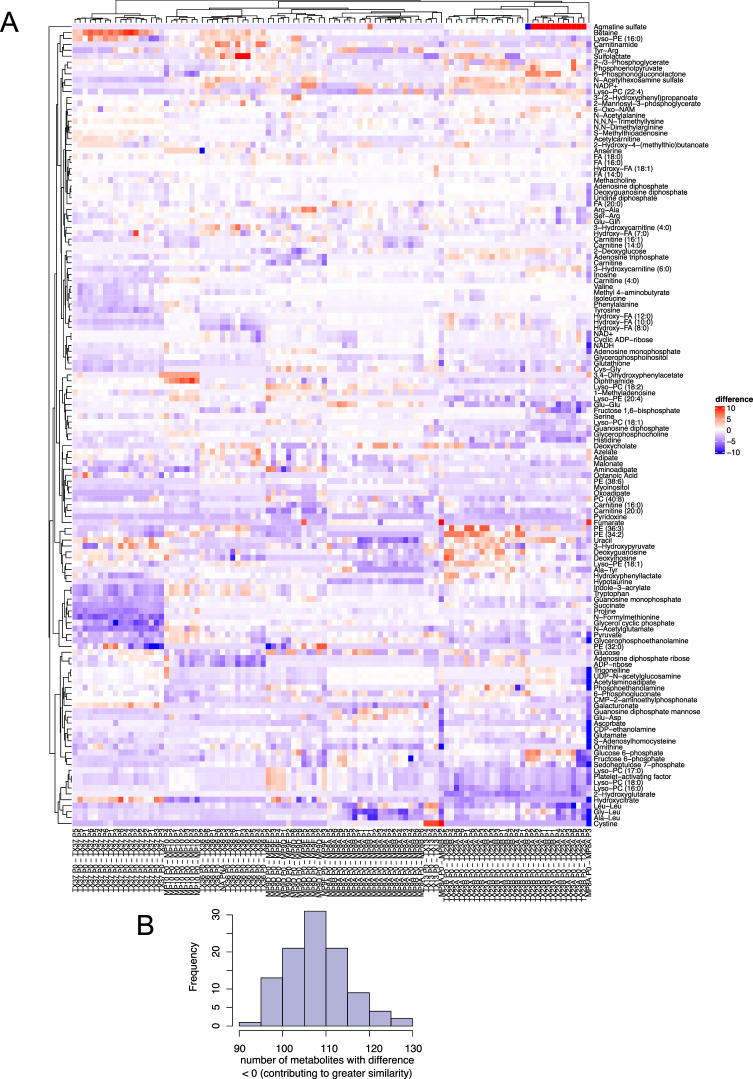
Metabolic contribution to patient-PDX matching. (**a**) Heatmap of metabolite-specific contributions to patient-PDX matching. The heatmap displays the Δdk values (difference between matched and average non-matched Euclidean distances) for each of the 143 metabolites across 171 matched patient-PDX pairs. Columns represent metabolites, while rows correspond to individual patient-PDX matches. Metabolites with negative Δdk (blue) contribute to greater similarity in matched pairs, while positive Δdk (red) indicate divergence. The variability in metabolite contributions across pairs reflects heterogeneity in patient-PDX metabolite profiles. (**b**) Histogram of metabolite contributions to similarity. The histogram summarizes the number of metabolites (Δdk < 0) contributing to greater similarity for each matched patient-PDX pair. The majority of matches exhibit 90–130 metabolites with Δdk < 0, indicating that most non-host-related metabolites play a role in driving patient-PDX resemblance.

To assess the contribution of host-independent metabolites to patient–PDX matching, we analysed the Euclidean distances between matched and non-matched pairs, focusing on the metabolite-specific contributions to similarity. For each metabolite, we calculated the difference in squared distances between matched and non-matched pairs (∆*d*_*k*_), as detailed in Methods. Metabolites with negative ∆*d*_*k*_ contributed to greater similarity between a tumour and the PDX derived from it. In most matches, 90–130 metabolites (out of 144) had ∆*d*_*k*_ < 0, meaning they enhanced similarity in matched patient–PDX pairs. Importantly, the specific metabolites driving similarity varied across matches (Extended Data Fig. 4a), reflecting heterogeneity in their contributions. On average, approximately 75% of the 144 metabolites exhibited greater similarity in matched pairs, emphasizing their collective role in driving patient-PDX resemblance (Extended Data Fig. 4b).

**Fig. 3:**
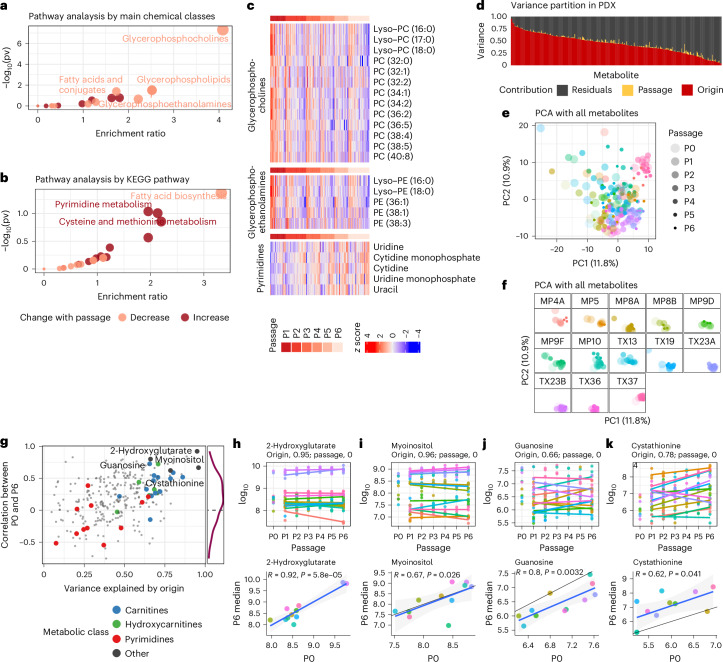
Metabolomic profiling of tumours from serially passaged melanoma PDXs. **a**,**b**, Pathway analysis of the metabolites changed by PDX passaging, as identified through multivariate analysis, from P1 to P6 (from *n* = 188 samples). For statistical analysis, a hypergeometric test was used without adjustment for multiple comparisons. pv, *P* value. **c**, Heatmap of altered metabolites in enriched pathways. Samples in columns are ordered by passage, and within each passage, by the average across selected metabolites, showing concordant changes of metabolites from the same class or pathway. **d**, Variance partition from a multivariate model predicting PDX metabolite levels by passage and origin. **e**,**f**, Principal component analysis (PCA) of patient and PDX samples by all metabolites, with all samples together (**e**) or separated by origin (**f**). **g**, Metabolites with high origin-specific variations (*x* axis) are also more concordant between patient tumour (P0) and late-passage PDX (P6) (*y* axis). **h**–**k**, Detailed examination of selected metabolites (also labelled in **g**). In each plot, the top panel shows the levels from P0 to P6, with linear regression lines fitted for each PDX line from P1 to P6. The variance contributed by origin or passage are given in the subtitle. Bottom, Pearson correlation between P0 and P6 samples and resulting *P* values. The black line denotes where *x* = *y*, and the blue line is a regression line from a linear fit. Colour denotes origin; see **f** for the colour key.

**Extended Data Fig. 5:**
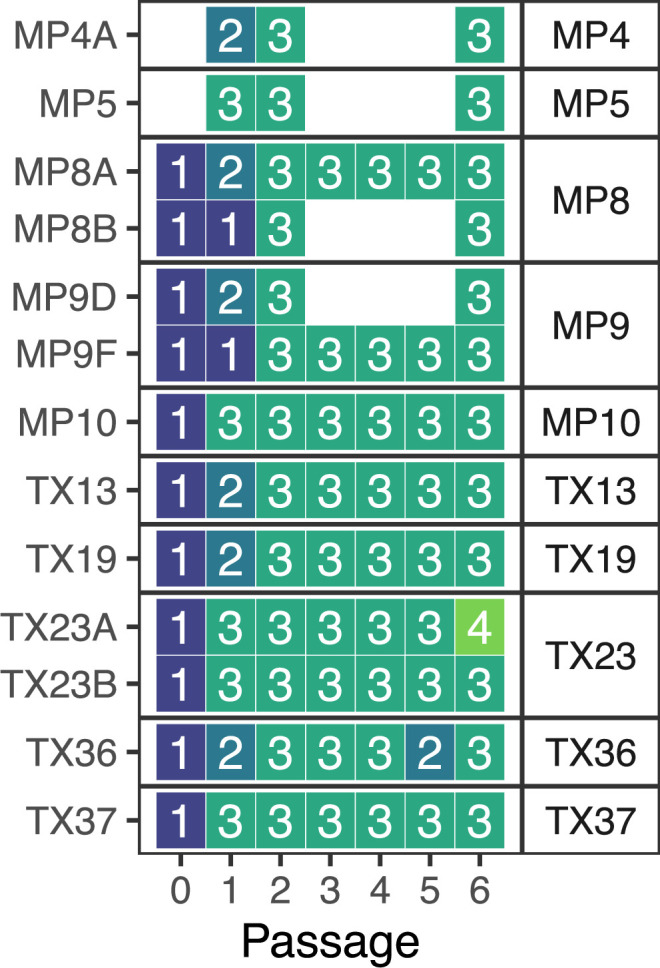
Tumor and PDX samples. Samples (n = 199) characterized by metabolomics in this study include patient tumor (passage 0) and serially passaged PDXs from 13 tumor fragments (left-side y-axis label) in 10 patients (right-side y-axis label).

In many pre-clinical studies using PDXs, later passage PDXs must be used to obtain enough mice to perform experiments. We therefore assessed metabolic durability over time to determine the extent of metabolic drift with serial passaging. We analysed a panel of 199 samples, including 13 PDX tumours passaged for 6 generations over as long as 3 years (Extended Data Fig. 5). We first performed a pathway analysis using metabolites that changed during passaging (Supplementary Table 6 and Fig. 3a,b). This revealed increased pyrimidine metabolites and decreased glycerolipids with serial passaging (Fig. 3a–c).

Despite these metabolites, which exhibited variation with repeated passage, most metabolic differences among PDXs still arose from the patient of origin. A multivariate model that predicts metabolite levels in PDXs on the basis of passage number and sample origin demonstrated that the impact of patient of origin was stronger than that of passage number (Fig. 3d). When an unsupervised analysis of all samples with all metabolites was performed across P1–P6 in 13 PDX models, the passage number was not the main source of variance in the top principal components (Fig. 3e). When each PDX was examined individually, most clustered together, with no clear separation by passage number (Fig. 3f). This reinforces the notion that the individual patient sample has a prominent impact on PDX metabolomics.

**Extended Data Fig. 6:**
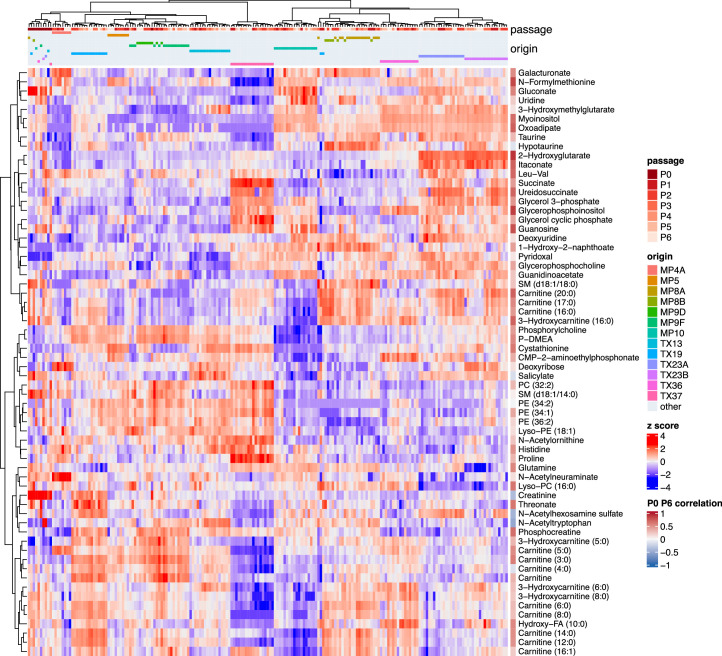
Signature metabolites cluster samples by lineage. Top metabolites that explain over 30% of the variance by origin and Pearson correlation > 0.3 between P0 and P6 samples (see Fig. 3d, n = 199 samples) were used in this heatmap. Metabolites and samples are hierarchically clustered into distinctive groups with PDXs from the same origin showing distinctive metabolic patterns. 10 of the 13 patient tumors formed a cluster separate from the rest of the PDX clusters. Note that PDX lines from the same patients - MP8A/B, MP9D/F, and TX23A/B generally cluster together.

**Extended Data Fig. 7:**
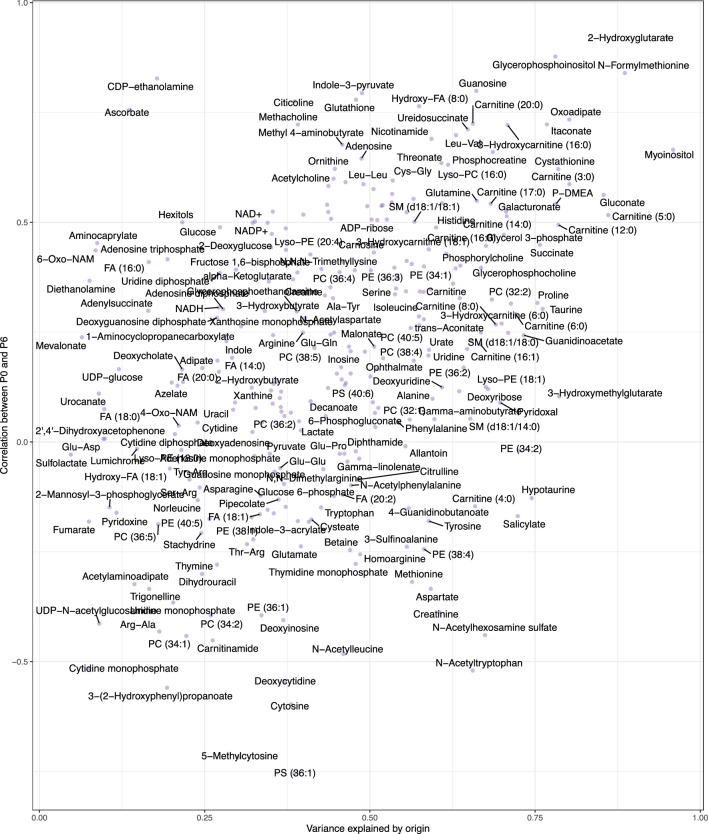
Plot of PDX metabolites based on their origin fidelity and stability. Metabolites are plotted based on origin-specific variations (x-axis) and concordance between patient tumor (P0) and late passage PDX (P6) (y-axis). (summarized from 11 patient tumors and 40 P6 PDX).

We also analysed the correlation between the patient tumour (P0) and the late-passage PDX (P6) across all metabolites, observing primarily positive correlations (Fig. 3g, *y* axis). Metabolites with large variations explained by patient origin (Fig. 3g, *x* axis) also tended to retain this correlation between P0 and P6 (Extended Data Fig. 6). Some classes of metabolites, such as carnitines and hydroxycarnitines, display high fidelity across passages. Other metabolites, like pyrimidines, have lower fidelity. A few examples of high-fidelity metabolites are highlighted and shown in Fig. 3h–k. 2-Hydroxyglutarate levels are distinctively higher in TX23A and TX23B (more than tenfold), and this is retained years later after six generations of tumour passaging (Fig. 3h). Similarly, myoinositol is distinct and stable across passages (Fig. 3i). We also found metabolites that demonstrated stability across P1–P6 PDX passages but markedly shifted from P0 (the patient) to P1. These include guanosine, which decreased between patient and PDX (Fig. 3j), and cystathionine, which increased (Fig. 3k). A mapping of all metabolites on the basis of their fidelity (*x* axis) and stability over time (*y* axis) in the PDX model system is available as a scientific resource (Extended Data Fig. 7 and Supplementary Table 4).

## Discussion

The goal of this study was to document the metabolic features of PDXs that do or do not maintain fidelity with donor tumours in patients. Previous work has uncovered metabolomic fingerprints among different melanoma PDX lines^27^, yet few studies have traced metabolic features from patient to PDX. We also used an intraoperative stable-isotope infusion protocol to examine consistency in glucose utilization between PDXs and patient tumours. Relative to other tumour types studied in humans, melanoma exhibits characteristics most similar to brain tumours, with glucose-derived carbons making large contributions to TCA cycle intermediates. Ongoing studies with [U-^13^C]glucose infusion will determine whether metabolic tracing data can predict outcomes or other relevant clinical features. If so, the large variance of labelling across patients with melanoma could represent an opportunity for biomarker development and patient stratification.

Of the variables examined in an extensive metabolomic analysis, host organism had the greatest influence on tumour metabolism, and very specific alterations were associated with diet and host physiology. These findings not only serve as validation of the data generation and analysis techniques, but also underscore the magnitude with which the environment impacts tumour metabolism. Although some alterations simply reflect host physiology, others might reflect a selection bias imposed on xenograft tumours. For example, PDX tumours consistently had higher labelling in glycolytic intermediates and increased levels of pyrimidine metabolites. The latter enrichment continued beyond the initial xenografting and throughout passaging. Whether this metabolic drift simply reflects ongoing expansion of the most proliferative clones or an adaptation related to NSG physiology is unclear. Similarly, we found that glycerophospholipids decreased across passages. The homogeneous fat sources used in our mouse-facility diets might contribute to this effect, but additional lipidomics analyses are required to test this hypothesis.

Despite some consistent and recognizable differences associated with xenografting, many metabolites and pathways were maintained throughout six generations. When removing species-specific metabolites, most melanoma PDXs had a ‘metabolic fingerprint’ that could be traced to the patient tumour. This identity was retained throughout passaging as PDXs from the same patient consistently clustered together. To contextualize this surprising finding, our unsupervised metabolomic analysis allowed 171 different PDX-derived samples to associate with any of 8 patient origins, and in 102 cases they associated with the single correct source. This occurred despite PDX-related heterogeneity, such as passage number or tumour size. Notably, many of the metabolites that demonstrated the strongest origin fidelity and stability were mitochondrial-associated metabolites (2-hydroxyglutarate, *n*-formylmethionine, oxoadipate and acylcarnitines).

Although some metabolic features are known to be sensitive to nutrient availability, others could be independent of surrounding environmental context and ‘fixed’ by driver mutations, mitochondrial disfunction or acquired pathway dependencies. These conserved metabolic features are important to identify because they could be clues to pathways that are less adaptable, and therefore more targetable in the context of oncologic therapeutics.

When considering metabolic conservation in the melanoma PDX model system, it is worth noting that tumours are implanted directly into the subcutaneous space of the mouse flank. Although many of the original tumours in this study came from melanoma tissues that had metastasized to lymph nodes or distant sites, we suspect that melanoma tumours are already metabolically ‘equipped’ to survive and proliferate in the skin. Other cancer types being established as PDXs in a non-orthotopic manner might feature additional metabolic variabilities that are not seen in the melanoma tumours owing to metabolic influences related to the tissue of origin^28^.

Through this analysis of 18 patient–PDX pairs and nearly 200 PDX melanomas passaged over time, we identified metabolites and pathways demonstrating both conservation and divergence. The findings lead us to advocate for a detailed evaluation of tumour metabolism in matched patient samples and PDXs prior to the use of PDXs for pre-clinical studies of tumour metabolism. Such an evaluation can highlight any potential metabolic differences and limitations that might influence the clinical relevance of pre-clinical studies.

## Methods

### Experimental model and study participant details

#### Human participants

This project was approved by the Institutional Review Board of UT Southwestern Medical Center and complied with all relevant ethical regulations. Recruitment of human participants and sample acquisition were done through the University of Texas Southwestern Medical Center Tissue Resource (STU102010-051, principal investigator (PI): C. Lewis), the University of Texas Southwestern Medical Center Human Melanoma Metabolism (HuMM) Biobank Project (STU102017-082, PI: J. Gill; STU052018-031, PI: J. Gill), and the University of Michigan (IRB MED no. 2004-0618, PI: A. Durham and MTA MMTA202002-0173, PI: J. Gill). Written informed consent was obtained from all participants. All adult patients undergoing standard-of-care surgical resection of melanoma with sufficient tissue for research were considered for enrolment. Those with poorly controlled diabetes were not eligible for ^13^C-glucose infusions. Demographics and pertinent clinical characteristics of patients are outlined in Supplementary Table 1.

#### Animals

All animal experiments were compliant with ethical regulations and performed in accordance with protocols approved by the Institutional Animal Care and Use Committee at the University of Texas Southwestern Medical Center (Protocol 2016-101360). Patient-derived melanoma tumours were injected into 4- to 8-week-old male and female NOD.CB17-*Prkdc*^scid^*Il2rg*^tm1Wjl^/SzJ (NSG) mice. No formal randomization algorithms were used and tumours were engrafted into randomly selected cages and processed in an arbitrary order. For all experiments, the maximum allowable tumour diameter was 2.5 cm and in no experiments was this exceeded. Mice were fed normal chow and ate ad-libitum. Mice were housed in barrier facilities managed by the UTSW Animal Resource Center. A standard white light cycle was used from 6:00AM to 5:59PM and a dark cycle was from 6:00PM to 5:59AM. Room humidity was maintained between 30 and 70% and temperature between 68 °F and 79°F.

#### Acquisition of human melanoma tumours

All human melanoma samples were obtained through surgical procedures that were a part of the patient’s standard of care. Portions of the tumour deemed by a pathologist to be available for research were provided for the studies. Samples were divided, and a portion of the tumour was immediately snap-frozen in liquid nitrogen in preparation for later metabolic processing. The other portion was placed in Leibovitz’s L-15 medium and placed on ice for processing and injection into mice, as described below. All melanoma samples (or their adjacent tissue from which they were taken) were reviewed by a pathologist for confirmatory diagnosis.

#### Generation of patient-derived xenografts

Melanoma tissue was transferred from Leibovitz’s L-15 medium into Kontes tubes. For larger tissues, samples were first quickly chopped with a razor blade on a sterile Petri dish before transfer; 1× Hank’s Balanced Salt Solution with 200 U ml^−1^ collagenase IV, 50 U ml^−1^, DNase and 5 mM CaCl_2_ was added to the Kontes tubes. Samples were then homogenized and digested for 20 min at 37 °C. Cells were then filtered through a 40-µm cell strainer and washed to obtain a single-cell suspension. Depending on the volume of the available tissue, the entire cell suspension was divided into three to five equal aliquots for injection into mice to establish the first generation of PDX tumours. For subsequent passages after successful engraftment, melanoma tissue was processed as described above, and 10,000 cells were aliquoted for each mouse. PDX melanoma cells in staining medium were mixed 1:1 with Matrigel for a final volume of 50 µl. This solution was drawn into syringes and injected subcutaneously into the right flank of the NSG mice. Subcutaneous tumours were measured weekly with calipers until tumours reached 2–2.5 cm in their greatest diameter, at which point mice were euthanized for endpoint tumour-metabolism assessment. To confirm accurate tumour identities after the passaging experiments, PDX tumours from passage 6 underwent geneprinting with the GenePrint® 10 System to ensure they matched the original patient and/or first passage as expected.

#### [^13^C]glucose isotope infusions

Patients receiving [^13^C]glucose infusions were generally fasted for 8–16 h, per the standard-of-care pre-operative protocol. A peripheral intravenous line was placed on the morning of the surgery to serve as a dedicated line for the [^13^C]glucose infusion. A sterile, pyrogen-free solution of 13.3% [U-^13^C]glucose in water was infused as a bolus of 8 g over 10 min, followed by 4 g h^−1^. Standard surgical procedures were followed for tumour resection. To assess fractional enrichment in plasma, peripheral blood samples were obtained before infusion, immediately after the bolus and every 30–60 min thereafter, using a different intravenous catheter than the one infusing the [U-^13^C]glucose. After melanoma tissue was obtained, the infusion was stopped. The average infusion duration was around 3 h. This infusion approach was consistent with previously described studies^13,15,16^.

Mouse infusions were conducted when PDX subcutaneous tumours were 2–2.5 cm in diameter. Mice were fasted for 16 h prior to infusion. Mice were anaesthetized and a 27-G catheter was placed in the lateral tail vein. [U-^13^C]glucose was infused as a bolus of 0.4125 mg g^−1^ of body mass over 1 min in 125 µl of normal saline, followed by continuous infusion of 0.008 mg g^−1^ body mass per minute for 3 h (in a volume of 150 µl h^−1^). To assess fractional enrichment in plasma, 20 µl of blood was collected retro-orbitally at baseline and after 30, 60, 120 and 180 min of infusion. At the end of the infusion, mice were euthanized, and tumours were collected and divided for sample processing (one portion for snap-freezing for metabolic analysis; another for digesting and passaging into additional generations of mice).

#### Sample processing

The mass of tissue samples processed generally ranged from 25 mg to 100 mg. For extraction of metabolites for metabolomic analysis, frozen tissues were homogenized manually with a pestle in an ice-cold mixture of methanol and water (80:20, vol/vol). After homogenization, samples were centrifuged at 13,000*g* for 15 min at 4 °C. Supernatants were transferred to a new Eppendorf tube and equivalent amounts (per BCA quantification) were dried down and resuspended in 80% acetonitrile for analysis. HILIC chromatographic separation of metabolites was performed through a Millipore ZIC-pHILIC column (5 µm, 2.1 × 150 mm) with a binary solvent system of 10 mM ammonium acetate in water (pH 9.8) and acetonitrile with a constant flow rate of 0.25 ml min^−1^. Metabolites were detected on a ThermoScientific QExactive HF-X hybrid quadrupole orbitrap high-resolution mass spectrometer (HRMS) coupled to a Vanquish UHPLC.

For extraction of metabolites for fractional enrichment analysis, frozen tissues were homogenized with a Qiagen TissueRuptor in an ice-cold mixture of methanol and water (80:20, vol/vol). Samples were then taken through three freeze–thaw cycles and centrifuged for 20 min at 16,000*g*. For an internal control, 1 µl of d27-myristic acid was added to the supernatant. Samples were evaporated and resuspended in 40 µl of anhydrous pyridine with 10 mg ml^−1^ methoxyamine. Samples were then mixed with 60 µl of *N*-(tert-butyldimethylsilyl)-*N*-methyltrifluoroacetamide (MTBSTFA) derivatization reagent and transferred to GC–MS vials. Samples were incubated for 1 h at 70 °C, and 1–2 µl was injected for analysis. Samples were analysed using GC–MS (an Agilent 6890 gas chromatograph coupled to an Agilent 5973.N mass selective detector or 7890 gas chromatograph coupled to an Agilent 5975C mass selective detector).

#### Mass spectrometry analysis

For metabolomics, metabolite identities were confirmed using three criteria: (1) the precursor ion *m/z* matched the theoretical mass predicted by the chemical formula within 5 ppm; (2) fragment ion spectra matched known metabolite fragments within 5 ppm; and (3) the metabolite retention time fell within 5% of the retention time of a purified standard run with the same method. Relative quantitation of the metabolites was done by integrating the chromatographic peak area of the precursor ion within a 5 ppm tolerance. To calculate relative abundance, the peak area was divided by the total ion chromatogram (TIC) for that sample.

For isotope tracing, unlabelled derivatized metabolite standards were previously used to generate an in-house library of mass spectra and served as references for the experimental metabolite peaks. The metabolite ion distribution and retention time of each metabolite peak were matched to ensure correct identification. The abundance of each metabolite ion was calculated and corrected for naturally abundant isotopes using a customized R script, found at the GitHub repository (https://github.com/wencgu/nac).

#### Histology and Ki-67 staining

Portions of tumour samples not used for metabolomics were placed in formalin and processed by either UTSW Department of Clinical Laboratory Services, the UTSW Tissue Management Shared Resource (TMSR), or the UTSW Histo Pathology Core. All histologic assessments were performed by a board-certified dermatopathologist (T.V.). Ki-67 staining was performed by the UTSW TMSR using the rabbit monoclonal antibody anti-Ki67 (Cell Signaling Technology, no. 9027). Immunohistochemical analysis was performed on a Leica Bond RX system. In brief, the slides were baked for 30 min at 60 °C, then deparaffinized before the antigen retrieval step. Heat-induced antigen retrieval was performed at pH 6 for 20 min. The tissue was incubated with a peroxidase block and then antibody (1:500) for 15 min. The staining was visualized using the Bond Polymer Refine detection system without the Bond post-primary reagent.

#### Materials availability

There are restrictions to the availability of human-derived specimens in this manuscript due to sharing limitations set by the Institutional Review Board. In cases in which samples may be shared, a materials-transfer agreement (MTA) must be obtained.

### Quantification and statistical analysis

#### Study design

Participant age and sex were reported by the human participants that were receiving melanoma surgical resections at our institution. Final sample size was dictated by the subsequent number of successfully xenografted tumours. All available samples were obtained and used for downstream analyses. No data were excluded. Although no statistical methods were used to pre-determine sample sizes, our sample sizes are similar to those reported using this infusion approach in previous studies^13,15,16^. For all analyses, data distribution was examined by density plot and was assumed to be normal, but this was not formally tested.

Data collection and analysis were not performed blind to the conditions of the experiment. Because analyses were performed computationally using predefined algorithms applied uniformly across samples, the potential for observer bias was effectively eliminated.

#### Analysis of isotope tracing data

To evaluate pyruvate dehydrogenase’s contribution to the TCA cycle, we used the ratio between citrate M+2 over pyruvate M+3 (CitM2/PyrM3 ratio) as a proxy and calculated this ratio for each sample. Samples from four origins—MP4A, MP8A, MP9D and MP10—were selected. Each set contained three types of samples—a single patient tumour (P0), multiple early-passage PDXs (P2/P3) and multiple late-passage PDXs (P6). A two-way ANOVA was conducted to explore the effects of ‘origin’ and ‘type’ on the CitM2/PyrM3 ratio. A two-sample *t*-test was also performed to evaluate differences between early- and late-passage PDXs. Results are visualized in Extended Data Figure 2a.

To evaluate the variability of additional labelled intermediates from the infusion experiments in patient tumour (P0) and early passage (P2/P3) PDXs, we included 3-phosphoglycerate M+3, pyruvate M+3, lactate M+3, alanine M+3, citrate M+2, glutamate M+2 (which exchanges with alpha-ketoglutarate M+2), succinate M+2, fumarate M+2, malate M+2 and aspartate M+2 (which exchanges with oxaloacetate M+2). The levels of these labelled intermediates were normalized to the glucose M+6 values in the same sample, and a two-way ANOVA was conducted to explore the effects of ‘origin’ and ‘type’ on these labelled markers, as visualized in Extended Data Figure 1b.

#### Metabolomics data processing

Metabolites not detected in more than 90% of the samples were excluded. Metabolites detected in all samples, with median log_10_ intensities between 5 and 10, were used to construct a normalization factor that represents total signal abundance in each sample. This normalization factor was computed by scaling individual values by the median intensity of each metabolite and subsequently taking the median of these ratios sample-wise. The normalized data were then log_10_-transformed, and missing values were imputed based on the minimum non-missing values within each metabolite. All processed metabolomics data are available in the source data file associated with the paper.

#### Analysis of 18 matched patient tumour and PDX samples (‘Batch 1’)

##### Metabolomics association with clinical features and sample types

For the metabolomics comparison of patient tumours and their PDX counterparts, 18 matched patient tumours and early-generation PDX samples were selected for analysis. Only one patient–PDX pair was selected per patient to prevent data skewing. Fifteen sample pairs were obtained from UTSW samples, outlined in Supplementary Table 1, and three sample pairs were obtained through an MTA with the University of Michigan.

Selected features including human or PDX (human/PDX) status, pigment status, *BRAF* mutation status (binary), sex and primary site, lymph node or metastasis designation were analysed through univariate statistical methods. For each selected feature, non-missing data were isolated, and a linear model was fit using the lmFit function from the limma package, followed by empirical Bayes moderation^29^. Significant metabolites were identified on the basis of adjusted *P* values (threshold < 0.05). Additionally, the variability of metabolite expression across the selected features was assessed to understand the variance component attributed to each factor. Likewise, we also explored the variance associated with human or PDX and pigment status in a multivariate linear model, as each of these features significantly associate a subset of metabolites in the univariate tests. This corresponds to data shown in Fig. 2b. From this multivariate model, we identified metabolites associated with human/PDX sample status to generate the volcano plot in Fig. 2c, with the fold change computed from converting data from the log_10_ to log_2_ scale. Selected metabolites were compared in Fig. 2d–g using the non-parametric Mann–Whitney test because some of the metabolites contain imputed values because of missing data.

##### Matching of patient and PDX samples

To compare the similarity between patient and PDX samples from the same origin versus different origins, all metabolites were *z*-transformed to compute Euclidean distance. The pairwise distance between a patient sample and a PDX sample was then compared with the Mann–Whitney test (Fig. 2k).

#### Analysis of patient tumour and matched serially passaged PDX samples (‘Batch 2’)

##### Matching of patient and PDX samples

As metabolomics data for the serially passaged PDX samples and the matched patient tumours were acquired in different batches, we performed a separate statistical test to identify metabolites that differ between the patient tumour and PDX. A linear mixed-effects model was employed using the lme function from the nlme package. This model was structured to include sample type (human tumour or PDX) as a fixed effect while accounting for the random effects associated with variations between origins. We determined *P* values for the sample type and identified metabolites with *P* values larger than 0.1 as species-agnostic metabolites. *Z*-transformed data of these species-agnostic metabolites were then used to compute Euclidean distance. When the distance between a PDX and a tumour from the same origin is the shortest among all pairwise PDX–tumour distances for the PDX, it is considered a correct match to the patient (Fig. 2l).

To determine what fraction of PDX metabolites were most closely matched to the correct patient tumour origin, we decomposed the total Euclidean distance between PDX and patient tumours into squared distances contributed by individual metabolites. This approach allowed us to identify the specific contributions of each metabolite to similarity or divergence.

### Pairwise Euclidean distance

The total Euclidean distance is defined as:

$${D}_{{total}}=\sqrt{\mathop{\sum }\limits_{k=1}^{{n}_{metabolites}}{({x}_{k}{-}{y}_{k})}^{2}}$$

where *x*_*k*_ is the value of metabolite *k* in the PDX sample, *y*_*k*_ is the value of metabolite *k* in the patient sample, and *n* is the total number of selected metabolites (144).

### Squared distance contributions

For each metabolite *k*, the squared distance contribution is:

$${d}_{k}={({x}_{k}{-}{y}_{k})}^{2}$$

To assess each metabolite’s role in similarity, we calculated the difference between ${d}_{k}^{{match}}$ (for the matched pair) and the mean squared distance ${\bar{d}}_{k}^{{non}-{match}}$ (for non-matched pairs):

$$\Delta {d}_{k}={d}_{k}^{\;\rm{match}}{-}{\bar{d}}_{k}^{\;\rm{non}-\rm{match}}$$

If (∆*d*_*k*_ < 0), the metabolite contributes to greater similarity between matched pairs.

If (∆*d*_*k*_ > 0), the metabolite contributes to divergence between matched pairs.

To quantify how many metabolites contribute to lower Euclidean distances (and hence higher similarity) between patient–PDX pairs, we counted the number of metabolites with a negative ∆*d*_*k*_, which is presented as a histogram (Extended Data Fig. 4b). A heatmap of ∆*d*_*k*_ across all 171 matches is presented in Extended Data Figure 4a.

#### Metabolomics associated with serial passaging or sample origin in PDX samples

To evaluate how origin and passage affect the metabolite profiles within the PDX samples from P1 to P6, we used lmFit from the limma package to fit a multivariate model including both features as fixed effects. On the basis of this model, we partitioned the overall variance observed in metabolites into components attributable to differences in passage numbers and tumour types, thereby enabling a deeper understanding of how these factors individually and jointly affect the metabolomic profiles. This corresponds to data shown in Fig. 3d. Pathway analysis was subsequently performed using metabolites identified as significantly altered owing to passaging effects in the multivariate analysis (Fig. 3a,b).

To evaluate fidelity of metabolites between patient tumour and late-passage PDXs, we computed Pearson correlation coefficients between P0 and P6 samples for each metabolite. Out of 305 metabolites, 102 were selected as characteristic metabolites on the basis of the following criteria: >30% variation explained by the origin and Pearson correlation > 0.3. These metabolites were used to cluster the samples in Extended Data Fig. 6. The P0 and P6 correlation and origin-associated variation were also used to identify high-fidelity metabolites reviewed in Fig. 3h–k.

#### Pathway analysis

Metabolic pathway enrichment analyses (in Figs. 2h and 3a,b) were performed using hypergeometric tests with metabolites detected in our custom metabolomics data as the background reference. The metabolic signature sets we queried include the Kyoto Encyclopedia of Genes and Genomes (KEGG), The Small Molecule Pathway Database (SMPDB) pathway libraries and ‘main class’ metabolite sets from RefMet^30^, adapted from MetaboAnalyst v.5.0 (ref. ^31^). Significant metabolites were visualized in heatmaps (Figs. 2i and 3c) using R package ComplexHeatmap^32^.

### Software and code

Patient tumour data were collected in and stored using REDCap (v.14.4.1). Data analysis in this study was performed using R v.4.2.3 (2023-03-15). The following packages were utilized: ComplexHeatmap (v.2.14.0), Hmisc (v.5.1-0), RColorBrewer (v.1.1-3), data.table (v.1.15.4), dplyr (v.1.1.4), ggbeeswarm (v.0.7.2), ggplot2 (v.3.5.0), ggpubr (v.0.6.0), ggrepel (v.0.9.5), ggside (v.0.3.1), limma (v.3.54.2), nlme (v.3.1-162), openxlsx (v.4.2.5.2), patchwork (v.1.2.0.9000), reshape2 (v.1.4.4), scales (v.1.3.0), stringr (v.1.5.1), tidyr (v.1.3.1), variancePartition (v.1.28.9) and viridis (v.0.6.3). Custom scripts for metabolomics analysis are available in the following GitHub repository: https://github.com/cailing20/Melanoma_PDX_metabolomics. A subset of graphs and data analysis were performed using GraphPad Prism (v.10.3.1).

### Reporting summary

Further information on research design is available in the Nature Portfolio Reporting Summary linked to this article.

## Supplementary information

Reporting Summary

Supplementary Tables 1–6

## Source data

Source Data Figs. 2 and 3 Metabolomics source data relevant to Figs. 2 and 3.

## Data Availability

Metabolomics and isotope-tracing data derived from human and PDX samples are available in the Supplementary Tables and Supplementary Data file associated with this manuscript. They are also publicly available from Dryad (10.5061/dryad.dncjsxm91). Source data are provided with this paper.

## Code availability

All original code has been deposited at https://github.com/cailing20/Melanoma_PDX_metabolomics and is publicly available.
