# Supplementary material for: Conservation and divergence of metabolic phenotypes between patient tumours and matched xenografts
Source: Nat Metab. 2025 Jul 29;7(8):1703–13. doi: 10.1038/s42255-025-01338-2 (PMC12373500; doi:10.1038/s42255-025-01338-2)
Supplement: Supplementary file 1 — Reporting Summary [file 42255_2025_1338_MOESM1_ESM.pdf]

nature portfolio

Corresponding author(s): Ralph J DeBerardinis, MD, PhD
Jennifer G. Gill, MD, PhD

Last updated by author(s): 05/28/2025

Reporting Summary

Nature Portfolio wishes to improve the reproducibility of the work that we publish. This form provides structure for consistency and transparency in reporting. For further information on Nature Portfolio policies, see our Editorial Policies and the Editorial Policy Checklist.

Statistics

For all statistical analyses, confirm that the following items are present in the figure legend, table legend, main text, or Methods section.

n/a	Confirmed	
☐	☒	The exact sample size (*n*) for each experimental group/condition, given as a discrete number and unit of measurement
☐	☒	A statement on whether measurements were taken from distinct samples or whether the same sample was measured repeatedly
☐	☒	The statistical test(s) used AND whether they are one- or two-sided<br>*Only common tests should be described solely by name; describe more complex techniques in the Methods section.*
☐	☒	A description of all covariates tested
☐	☒	A description of any assumptions or corrections, such as tests of normality and adjustment for multiple comparisons
☐	☒	A full description of the statistical parameters including central tendency (e.g. means) or other basic estimates (e.g. regression coefficient) AND variation (e.g. standard deviation) or associated estimates of uncertainty (e.g. confidence intervals)
☐	☒	For null hypothesis testing, the test statistic (e.g. *F*, *t*, *r*) with confidence intervals, effect sizes, degrees of freedom and *P* value noted<br>*Give P values as exact values whenever suitable.*
☒	☐	For Bayesian analysis, information on the choice of priors and Markov chain Monte Carlo settings
☐	☒	For hierarchical and complex designs, identification of the appropriate level for tests and full reporting of outcomes
☐	☒	Estimates of effect sizes (e.g. Cohen's *d*, Pearson's *r*), indicating how they were calculated

Our web collection on statistics for biologists contains articles on many of the points above.

Software and code

Policy information about availability of computer code

Data collection	Patient tumor data was collected in and stored using REDCap 14.4.1.
Data analysis	Data analysis in this study was performed using R version 4.2.3 (2023-03-15). The following packages were utilized: ComplexHeatmap (2.14.0), Hmisc (5.1-0), RColorBrewer (1.1-3), data.table (1.15.4), dplyr (1.1.4), ggbeeswarm (0.7.2), ggplot2 (3.5.0), ggpubr (0.6.0), ggrepel (0.9.5), ggside (0.3.1), limma (3.54.2), nlme (3.1-162), openxlsx (4.2.5.2), patchwork (1.2.0.9000), reshape2 (1.4.4), scales (1.3.0), stringr (1.5.1), tidyr (1.3.1), variancePartition (1.28.9), viridis (0.6.3). Custom scripts for metabolomics analysis are available in the following GitHub repository: https://github.com/cailing20/Melanoma_PDX_metabolomics. A subset of graphs and data analysis were performed using GraphPad Prism 10.3.1.

For manuscripts utilizing custom algorithms or software that are central to the research but not yet described in published literature, software must be made available to editors and reviewers. We strongly encourage code deposition in a community repository (e.g. GitHub). See the Nature Portfolio guidelines for submitting code & software for further information.

Data

Policy information about availability of data

All manuscripts must include a data availability statement. This statement should provide the following information, where applicable:

- Accession codes, unique identifiers, or web links for publicly available datasets
- A description of any restrictions on data availability
- For clinical datasets or third party data, please ensure that the statement adheres to our policy

Metabolomics and isotope tracing data derived from human and PDX samples are available in the Supplementary Tables and data files associated with this manuscript. They are also available from Dryad (https://doi.org/10.5061/dryad.dncjsxm91).

For pathway analysis, the metabolic signature sets we queried include the Kyoto Encyclopedia of Genes and Genomes (KEGG), The Small Molecule Pathway Database (SMPDB) pathway libraries and "main class" metabolite sets from RefMet, adapted from MetaboAnalyst 5.0.

Research involving human participants, their data, or biological material

Policy information about studies with human participants or human data. See also policy information about sex, gender (identity/presentation), and sexual orientation and race, ethnicity and racism.

Reporting on sex and gender	Human participants were recruited to the study independent of their sex or gender. The sex of human participants is documented in Table 1 and was determined based on self-reporting. Sex was one of the covariates analyzed in Figure 2A and was not a source of significant metabolic variation across tumors.
Reporting on race, ethnicity, or other socially relevant groupings	Human participants were recruited to the study independent of their race, ethnicity, or other socially relevant groupings. These variables are not reported or utilized as covariates in these studies.
Population characteristics	All human participants with melanoma tissue available for research were eligible for inclusion in these studies. Relevant patient and tumor data are included in Table 1.
Recruitment	Patients undergoing melanoma surgery and with tissue available for research were recruited for participation. Unbiased approaches were used to identify patients including screening of the electronic health record for key diagnoses codes and through the UTSW Multidisciplinary Melanoma Tumor Board.
Ethics oversight	The UTSW Institutional Review Board approved this project. IRB approval numbers are indicated in the manuscript.

Note that full information on the approval of the study protocol must also be provided in the manuscript.

Field-specific reporting

Please select the one below that is the best fit for your research. If you are not sure, read the appropriate sections before making your selection.

☒ Life sciences ☐ Behavioural & social sciences ☐ Ecological, evolutionary & environmental sciences

For a reference copy of the document with all sections, see nature.com/documents/nr-reporting-summary-flat.pdf

Life sciences study design

All studies must disclose on these points even when the disclosure is negative.

Sample size	Sample size was dictated by the number of human participants that were receiving melanoma surgical resections at our institution and the subsequent number of successfully xenografted tumors. All available samples were obtained and used for downstream analyses.
Data exclusions	No data was excluded.
Replication	True replication is not possible in the human arm of the study due to each tumor sample originating from one unique patient. However, during the PDX portion of this study, reproducibility was built into the study design with sampling from multiple xenografted mice per passage per tumor (see Figure S2).
Randomization	This study does not involve allocation into specific experimental groups as it is observational in nature (eg assessing the differences across variables such as host species, pigmentation, etc. that are naturally occurring and fixed).
Blinding	Blinding was not relevant to this study because all analyses were performed computationally using algorithms applied uniformly across samples. The outcomes were determined by objective parameters derived from sample annotations (e.g. sample identity, mutation status, etc), without subjective interpretation influencing data processing or outcome classification. Because of the automated and annotation-driven nature of the analytic pipeline, the potential for observer bias was largely mitigated.

Reporting for specific materials, systems and methods

We require information from authors about some types of materials, experimental systems and methods used in many studies. Here, indicate whether each material, system or method listed is relevant to your study. If you are not sure if a list item applies to your research, read the appropriate section before selecting a response.

Materials & experimental systems

n/a	Involved in the study
☐	☒ Antibodies
☒	☐ Eukaryotic cell lines
☒	☐ Palaeontology and archaeology
☐	☒ Animals and other organisms
☒	☐ Clinical data
☒	☐ Dual use research of concern
☒	☐ Plants

Methods

n/a	Involved in the study
☒	☐ ChIP-seq
☒	☐ Flow cytometry
☒	☐ MRI-based neuroimaging

Antibodies

Antibodies used	rabbit mAb Anti-Ki67 (Cell Signaling Technology, #9027)
Validation	This antibody has been validated by the manufacturer for use in immunohistochemistry against human Ki-67 (as used in this manuscript). Additionally, it has been used widely with over 450 citations in the literature.

Animals and other research organisms

Policy information about studies involving animals; ARRIVE guidelines recommended for reporting animal research, and Sex and Gender in Research

Laboratory animals	All mice used in these experiments were NOD.CB17-PrkdcscidIl2rgtm1Wjl/SzJ (NSG) mice injected at an age of 4- to 8-weeks old. Housing conditions are described in the manuscript.
Wild animals	No wild animals were used.
Reporting on sex	Sex was not considered in the study design or methods. No formal randomization algorithms were used and tumors were engrafted into randomly selected cages of either sex and processed in an arbitrary order.
Field-collected samples	No field-collected samples were used.
Ethics oversight	All animal experiments were approved by the Institutional Animal Care and Use Committee at the University of Texas Southwestern Medical Center (Protocol 2016-101360).

Note that full information on the approval of the study protocol must also be provided in the manuscript.

Plants

Seed stocks	N/A
Novel plant genotypes	N/A
Authentication	N/A
